# Time to embrace Dr. Google?

**DOI:** 10.1503/cmaj.1095935

**Published:** 2021-04-19

**Authors:** Diana Duong

**Affiliations:** *CMAJ*

For years, many health professionals have discouraged patients from searching online for information about symptoms, arguing it leads to unnecessary worry and strain on health systems. But some clinicians are rethinking that stance as new research suggests that fears about Dr. Google may be overblown.

A study of 5000 Americans by Harvard Medical School researchers found that searching symptoms online modestly boosted patients’ ability to accurately diagnose health issues without increasing their anxiety or misleading them to seek care inappropriately.

Participants reviewed clinical cases and imagined that a family member was experiencing the symptoms described. Researchers asked them to come up with a diagnosis and select one of four triage options, ranging from allowing the issue to resolve on its own to calling 911. Then, the participants repeated the task using the Internet to inform their decisions. They also rated their anxiety levels and confidence in their responses.

The study results, published in *JAMA Network Open*, showed that consulting Google increased the accuracy of participants’ proposed diagnoses from 50% to 54%, but did not affect their triage choices or increase their anxiety.

The study authors acknowledged that the participants may have made different choices in real life, but noted that their anxiety levels increased with the severity of the cases, “suggesting that they were internalizing the cases appropriately.”

The authors also admitted that “any benefit of an Internet search was small,” possibly because of the poor quality of information available online.

Even so, they concluded, “the results of this survey study challenge the common belief among clinicians and policy-makers that using the Internet to search for health information is harmful.”

**Figure:**
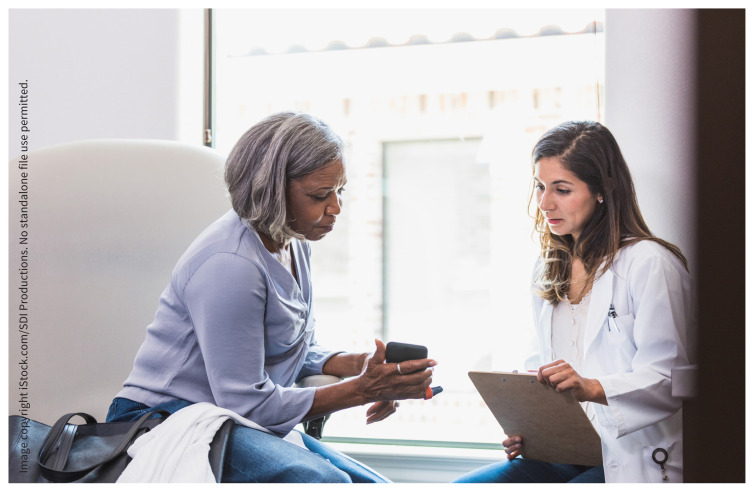
Searching symptoms online may not be as harmful as some feared.

Image copyright iStock.com/SDI Productions. No standalone file use permitted.

Other recent studies have called into question the assumption that Googling symptoms will send patients into an anxious spiral, dubbed “cyberchondria.” In one randomized controlled trial, patients waiting in an emergency department reported no changes in anxiety after Googling their symptoms.

Attitudes among clinicians appear to be shifting, too. Some have argued that discouraging patients from online research will only lead them to avoid discussing misinformation with their doctors. Others contend that clinicians should welcome questions and challenges from their patients rather than judging them.

Dr. Ritika Goel, a Toronto-based family physician, said clinicians must “embrace the reality that more and more patients will come to us having looked for information on their symptoms, potential diagnoses, and treatments.”

“This by definition disrupts the traditional power dynamics between patient and physician,” Goel said. “But we can see this as an opportunity to be our patients’ guides, point them to good information sources, and empower their role in health care decision-making.”

That starts with ensuring that patients don’t feel judged or shamed for looking up information — especially if their conclusions are inaccurate. “I would much rather know what my patients are reading and concerned about than have them feel they need to hide this information from me,” Goel said.

Physicians can also proactively recommend reputable resources when discussing topics with patients. For example, Goel regularly refers patients to the Society of Obstetricians and Gynaecologists of Canada’s *Sex & U* website for information on contraception.

Although companies like Google have improved the quality of health-related searches, many patients may still need help separating credible results from misinformation.

According to Dr. Eric Cadesky, a Vancouver-based family physician, refusing to engage with the information patients consume online is counterproductive. He argues that digital literacy is an important part of health.

“We have reached a point where if we don’t teach patients to seek good-quality information they will have harmful lies, unscientific myths, and conspiracy theories pushed to them,” Cadesky says. “Doctors need to have resources to answer patients’ questions, otherwise the void of information will be filled by bad actors.”

